# Novel Effective Bacillus cereus Group Species “*Bacillus clarus*” Is Represented by Antibiotic-Producing Strain ATCC 21929 Isolated from Soil

**DOI:** 10.1128/mSphere.00882-20

**Published:** 2020-11-04

**Authors:** Marysabel Méndez Acevedo, Laura M. Carroll, Manjari Mukherjee, Emma Mills, Lingzi Xiaoli, Edward G. Dudley, Jasna Kovac

**Affiliations:** a Department of Food Science, The Pennsylvania State University, University Park, Pennsylvania, USA; b Department of Natural Sciences, University of Puerto Rico in Aguadilla, Aguadilla, Puerto Rico, USA; c Department of Food Science, Cornell University, Ithaca, New York, USA; University of California, Davis

**Keywords:** *Bacillus cereus* group, *Bacillus cereus sensu lato*, *Bacillus clarus*, antibiotic production, cerexin A, cytotoxicity, enterotoxins, novel species, taxonomy, whole-genome sequencing

## Abstract

Gram-positive, spore-forming members of the Bacillus cereus group species complex are widespread in natural environments and display various degrees of pathogenicity. Recently, B. cereus group strain Bacillus mycoides Flugge ATCC 21929 was found to represent a novel lineage within the species complex, sharing a relatively low degree of genomic similarity with all B. cereus group genomes (average nucleotide identity [ANI] < 88). ATCC 21929 has been previously associated with the production of a patented antibiotic, antibiotic 60-6 (i.e., cerexin A); however, the virulence potential and growth characteristics of this lineage have never been assessed. Here, we provide an extensive genomic and phenotypic characterization of ATCC 21929, and we assess its pathogenic potential *in vitro.* ATCC 21929 most closely resembles Bacillus paramycoides NH24A2^T^ (ANI and *in silico* DNA-DNA hybridization values of 86.70 and 34.10%, respectively). Phenotypically, ATCC 21929 does not possess cytochrome *c* oxidase activity and is able to grow at a range of temperatures between 15 and 43°C and a range of pH between 6 and 9. At 32°C, ATCC 21929 shows weak production of diarrheal enterotoxin hemolysin BL (Hbl) but no production of nonhemolytic enterotoxin (Nhe); at 37°C, neither Hbl nor Nhe is produced. Additionally, at 37°C, ATCC 21929 does not exhibit cytotoxic effects toward HeLa cells. With regard to fatty acid composition, ATCC 21929 has iso-C17:0 present in highest abundance. Based on the characterization provided here, ATCC 21929^T^ (= PS00077A^T^ = PS00077B^T^ = PSU-0922^T^ = BHP^T^) represents a novel effective B. cereus group species, which we propose as effective species “Bacillus clarus.”

**IMPORTANCE** The B. cereus group comprises numerous closely related lineages with various degrees of pathogenic potential and industrial relevance. Species-level taxonomic classification of B. cereus group strains is important for risk evaluation and communication but remains challenging. Biochemical and phenotypic assays are often used to assign B. cereus group strains to species but are insufficient for accurate taxonomic classification on a genomic scale. Here, we show that antibiotic-producing ATCC 21929 represents a novel lineage within the B. cereus group that, by all metrics used to delineate prokaryotic species, exemplifies a novel effective species. Furthermore, we show that ATCC 21929 is incapable of producing enterotoxins Hbl and Nhe or exhibiting cytotoxic effects on HeLa cells at human body temperature *in vitro*. These results provide greater insight into the genomic and phenotypic diversity of the B. cereus group and may be leveraged to inform future public health and food safety efforts.

## Abstract (precis)

The B. cereus group comprises numerous closely related lineages with various degrees of pathogenic potential and industrial relevance. Species-level taxonomic classification of B. cereus group strains is important for risk evaluation and communication but remains challenging. Biochemical and phenotypic assays are often used to assign B. cereus group strains to species but are insufficient for accurate taxonomic classification on a genomic scale. Here, we show that antibiotic-producing ATCC 21929 represents a novel lineage within the B. cereus group that, by all metrics used to delineate prokaryotic species, exemplifies a novel effective species. Furthermore, we show that ATCC 21929 is incapable of producing enterotoxins Hbl and Nhe or exhibiting cytotoxic effects on HeLa cells at human body temperature *in vitro*. These results provide greater insight into the genomic and phenotypic diversity of the B. cereus group and may be leveraged to inform future public health and food safety efforts.

## OBSERVATION

The Bacillus cereus group is a complex of closely related, spore-forming, facultatively anaerobic bacterial species. Currently, the B. cereus group contains 19 published species: *albus* (1), *anthracis* (2), *cereus sensu stricto* (2), *cytotoxicus* (3), *fungorum* (4), *luti* (1), *mobilis* (1), *mycoides* (2), *nitratireducens* (1), *pacificus* (1), *paramycoides* (1), *paranthracis* (1), *proteolyticus* (1), *pseudomycoides* (5), *thuringiensis* (2), *toyonensis* (6), *tropicus* (1), *weihenstephanensis* (7), and *wiedmannii* (8). Additionally, the group currently contains three effective species: “*bingmayongensis*” (9), “*gaemokensis*” (10), and “*manliponensis*” (11).

We recently queried all publicly available B. cereus group genomes (12) and identified a singleton strain, B. mycoides Flugge ATCC 21929 (referred to here as ATCC 21929), that shared a relatively low degree of genomic similarity with all other genomes. Prior publications indicated that ATCC 21929 had been isolated from soil in Papua New Guinea and is able to produce a patented antibiotic compound, antibiotic 60-6 (also known as cerexin A), which is active against Gram-positive pathogens (13, 14). Here, a polyphasic approach that integrated genomic and phenotypic analyses was used to characterize ATCC 21929, a representative of novel effective species “Bacillus clarus.”

## 

### ATCC 21929 belongs to a novel B. cereus group genomospecies.

The ATCC 21929 genome was resequenced to confirm its identity (NCBI accession no. QVOD00000000; see Text S1 in the supplemental material). The original genome (NCBI RefSeq accession no. GCF_000746925.1) (14) was used in subsequent analyses.

10.1128/mSphere.00882-20.1 TEXT S1 Detailed descriptions of all methods and additional references. Download Text S1, PDF file, 0.2 MB. Copyright © 2020 Méndez Acevedo et al. 2020 Méndez Acevedo et al. This content is distributed under the terms of the Creative Commons Attribution 4.0 International license.

**FIG 1:**
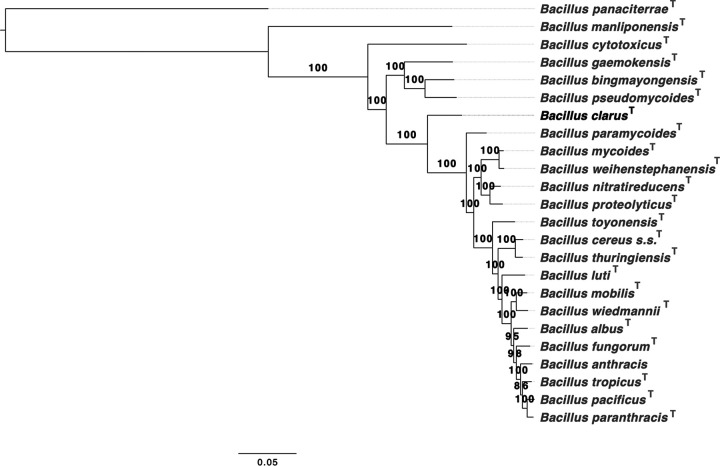
Maximum likelihood phylogeny constructed using concatenated amino acid sequences derived from the type strain/representative genomes of the 22 published and effective B. cereus group species (gray font), outgroup genome Bacillus panaciterrae^T^ (itself not a member of the B. cereus group; gray font), and novel effective B. cereus group species “*B. clarus*” strain ATCC 21929^T^ (black font). *B. panaciterrae*^T^ was used to root the phylogeny, and branch lengths are reported in substitutions per site. Node labels correspond to branch support percentages obtained using 1,000 replicates of the ultrafast bootstrap approximation. OrthoFinder (19) was used to identify orthologues among all genomes and produce the amino acid sequence alignment, and IQ-TREE (20) was used to construct the phylogeny. *s.s.*, *sensu stricto*.

Despite sharing 99.8% and 100% 16S rRNA gene sequence similarity and coverage with Bacillus tropicus (Fig. S1 and Text S1), respectively, ATCC 21929 shared <88 average nucleotide identity (ANI) with all B. cereus group genomes (accessed 19 November 2018; calculated using FastANI version 1.0) (12). Based on ANI and *in silico* DNA-DNA hybridization (DDH) values calculated between all published and effective B. cereus group species type strain/representative genomes, as well as the whole-genome phylogeny, ATCC 21929 most closely resembled Bacillus paramycoides (Fig. 1, Table 1, and Table S1). ATCC 21929 shared 86.70 ANI with *B. paramycoides* (calculated using JSpeciesWS, http://jspecies.ribohost.com/jspeciesws/, accessed 15 July 2020; Table 1 and Table S1) (15), which is well below all proposed species thresholds for the B. cereus group (i.e., 92.5 to 96 ANI) (1, 3, 6, 8, 12). The *in silico* DDH value (calculated using the Genome-to-Genome Distance Calculator [GGDC], https://ggdc.dsmz.de/, accessed 15 July 2020) (16) shared by ATCC 21929 and *B. paramycoides* was 34.10% (95% confidence interval, 31.60 to 36.60%; Table 1 and Table S1), with 0.48% probability that the DDH value is greater than the 70% species threshold (Table S1) (16). Based on these results, ATCC 21929 is a member of the B. cereus group but is not a member of any published (valid) or effective species.

**TABLE 1:** List of B. cereus group strains used in this study^*a*^

Contemporary *Bacillus* species name	Strain	Species status^*b*^	RefSeq accession no.	*panC* group (% homology)^*c*^	MLST ST^*d*^	JSpeciesWS ANIb^*e*^	GGDC DDH^*f*^ (%)
None (“*B. clarus*”)	ATCC 21929	Novel effective	GCF_000746925.1	VI (91.71)	1834^*g*^	100.00	100.00
*B. albus*	N35-10-2	Published	GCF_001884185.1	II (95.17)	775	84.83	30.70
B. anthracis	Ames	Published	GCF_000007845.1	III (100.00)	1	85.01	31.00
B. cereus *sensu stricto*	ATCC 14579	Published	GCF_000007825.1	IV (100.00)	921	84.84	30.90
*B. cytotoxicus*	NVH 391-98	Published	GCF_000017425.1	VII (100.00)	NA	81.36	26.90
*B. fungorum*^*h*^	17-SMS-01	Published	GCF_002746455.1	II (98.01)	NA	84.96	31.00
*B. luti*	TD41	Published	GCF_001884105.1	II (93.75)	764	85.09	31.00
*B. mobilis*	0711P9-1	Published	GCF_001884045.1	II (97.71)	784	84.81	30.70
B. mycoides	DSM 2048	Published	GCF_000003925.1	VI (100.00)	116	86.31	33.20
*B. nitratireducens*	4049	Published	GCF_001884135.1	II (96.02)	769	86.17	32.60
*B. pacificus*	EB422	Published	GCF_001884025.1	III (100.00)	32	84.92	30.60
*B. paramycoides*	NH24A2	Published	GCF_001884235.1	VI (94.03)	780	86.70	34.10
*B. paranthracis*	Mn5	Published	GCF_001883995.1	III (99.72)	761	84.90	30.60
*B. proteolyticus*	TD42	Published	GCF_001884065.1	II (94.89)	765	86.23	32.70
B. pseudomycoides	DSM 12442	Published	GCF_000161455.1	I (100.00)	83	83.90	30.20
B. thuringiensis	ATCC 10792	Published	GCF_002119445.1	IV (100.00)	10	84.83	30.70
*B. toyonensis*	BCT-7112	Published	GCF_000496285.1	V (100.00)	111	85.38	32.10
*B. tropicus*	N24	Published	GCF_001884035.1	III (98.01)	771	85.02	30.70
B. weihenstephanensis	WSBC 10204	Published	GCF_000775975.1	VI (100.00)	196	86.14	32.70
*B. wiedmannii*	FSL W8-0169	Published	GCF_001583695.1	II (99.43)	1081	85.00	31.00
“*B. bingmayongensis*”	FJAT-13831	Effective	GCF_000299035.1	NA (no leader sequence found) (0)	763	83.67	29.50
“*B. gaemokensis*”	JCM 15801	Effective	GCF_000712615.1	NA (no leader sequence found) (0)	768	84.21	29.40
“*B. manliponensis*”	JCM 15802	Effective	GCF_000712595.1	NBc (not B. cereus group) (28.00)	NA	77.01	23.30

a See Table S1 for an extended version of this table.

b Novel effective, “*B. clarus*” strain ATCC 21929 characterized here; published, one of 19 published B. cereus group species; effective, previously proposed in a peer-reviewed publication as a potential member of the B. cereus group but not officially recognized as a published species.

c *panC* group (I to VII) assigned using the Sym’Previus B. cereus group *panC* group assignment Web server (https://tools.symprevius.org/Bcereus/; accessed 16 July 2020) and the seven-group framework proposed by Guinebretiere et al. (21).

d Multilocus sequence typing (MLST) sequence type (ST); assigned *in silico* using BTyper version 2.3.2 and the B. cereus seven-gene MLST scheme available in PubMLST; NA, not assigned.

e Average nucleotide identity BLAST (ANIb) values calculated using the JSpecies Web server (JSpeciesWS, accessed 15 July 2020; http://jspecies.ribohost.com/jspeciesws/); novel effective species “*B. clarus*” strain ATCC 21929 was used as a query, and the listed genome was used as a reference. Genomospecies thresholds of 92.5 to 96 ANI have been proposed for the B. cereus group (1, 3, 6, 8, 12), and two genomes are considered to be members of the same genomospecies if they share an ANI value above this threshold.

f *In silico* DNA-DNA hybridization (DDH) values calculated using the Genome-to-Genome Distance Calculator (GGDC; accessed 15 July 2020; http://ggdc.dsmz.de/), Formula 2 (i.e., the formula recommended by GGDC); novel effective species “*B. clarus*” strain ATCC 21929 was used as a query, and the listed genome was used as a reference. Two genomes that share > 70% DDH are often considered to be members of the same species (16).

g “*B. clarus*” strain ATCC 21929 initially could not be assigned to any known PubMLST ST; its submission to PubMLST under ID number 2468 resulted in novel ST 1834.

h *B. fungorum*, a novel species proposed in March 2020 (4), was not included in the phenotypic portion of this study.

10.1128/mSphere.00882-20.2 FIG S1 Maximum likelihood phylogeny constructed using 16S rRNA gene sequences of type/representative strains for the 22 published and effective B. cereus group species (gray labels) and novel effective B. cereus group species “*B. clarus*” strain ATCC 21929^T^ (black label). The phylogeny is rooted at the midpoint, and branch lengths are reported in substitutions per site. Node labels correspond to branch support percentages obtained using 1,000 replicates of the ultrafast bootstrap approximation. 16S rRNA gene sequences were extracted from all 23 genomes using BTyper and aligned using MUSCLE. IQ-TREE was used to construct the phylogeny from the resulting alignment. Download FIG S1, PDF file, 0.1 MB. Copyright © 2020 Méndez Acevedo et al. 2020 Méndez Acevedo et al. This content is distributed under the terms of the Creative Commons Attribution 4.0 International license.

10.1128/mSphere.00882-20.5 TABLE S1 B. cereus group genomes used in this study (*n *= 23). Download Table S1, XLSX file, 0.02 MB. Copyright © 2020 Méndez Acevedo et al. 2020 Méndez Acevedo et al. This content is distributed under the terms of the Creative Commons Attribution 4.0 International license.

### ATCC 21929 does not produce Hbl or Nhe at human body temperature *in vitro*.

**FIG 2:**
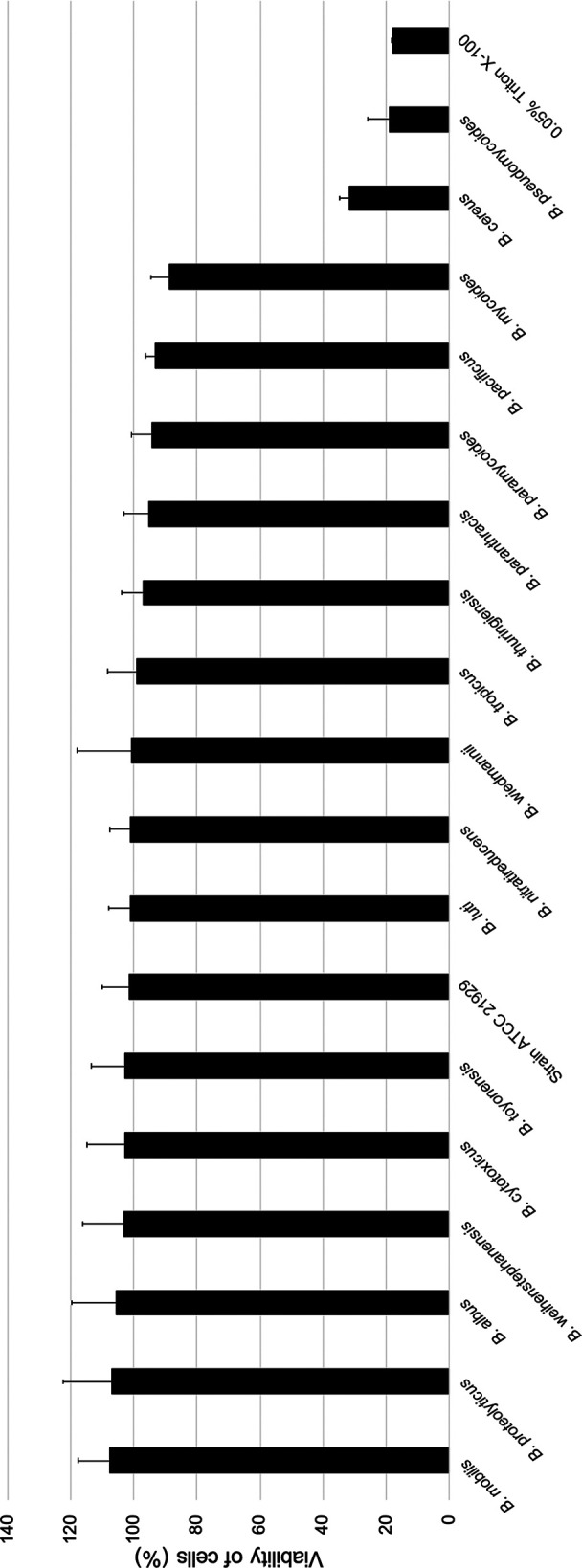
Percent viability of HeLa cells when treated with supernatants of novel effective B. cereus group species “*B. clarus*” strain ATCC 21929^T^ or one of 17 published B. cereus group species type strains, as determined by the WST-1 assay. Viability was calculated as the ratio of corrected absorbance of suspension when HeLa cells were treated with supernatants to the ratio of corrected absorbance of suspension when HeLa cells were treated with BHI (i.e., negative control), converted to percentages. The columns represent the mean viabilities, while the error bars represent standard deviations for 12 technical replicates.

Thirteen biosynthetic gene clusters (BGCs) were detected in the ATCC 21929 genome (Table 2) using antiSMASH (Text S1) (17). Genes encoding enterotoxins hemolysin BL (Hbl; *hblABCD*) and nonhemolytic enterotoxin (Nhe; *nheABC*) were additionally detected (Text S1). The Duopath Cereus Enterotoxins kit (Merck; Text S1) confirmed weak production of Hbl at 32°C but no production of Nhe. At 37°C, neither Hbl nor Nhe was produced. ATCC 21929 additionally demonstrated a lack of cytotoxic activity toward HeLa cells at 37°C (Fig. 2 and Text S1).

**TABLE 2:** Biosynthetic gene clusters (BGCs) identified in the genome of “*B. clarus*” strain ATCC 21929^*a*^

Region ID	Contig	antiSMASH type(s)^*b*^	Position		Most similar known BGC^*c*^	MIBiG biosynthetic class(es)^*c*^^,^^*d*^	Similarity (%)^*c*^
			From	To			
8.1	NZ_JMQC01000008.1	Bacteriocin	236159	247095	NA	NA	NA
8.2	NZ_JMQC01000008.1	Bacteriocin	275152	284251	NA	NA	NA
8.3	NZ_JMQC01000008.1	Terpene	401148	423004	NA	NA	NA
8.4	NZ_JMQC01000008.1	Lasso peptide	2059397	2083314	Paeninodin	RiPP	100
8.5	NZ_JMQC01000008.1	Bacteriocin	3248599	3257252	NA	NA	NA
8.6	NZ_JMQC01000008.1	LAP, bacteriocin	3525556	3549093	NA	NA	NA
8.7	NZ_JMQC01000008.1	NRPS	4544696	4594452	Bacillibactin	NRP	46
8.8	NZ_JMQC01000008.1	Beta-lactone	4710276	4735514	Fengycin	NRP	40
8.9	NZ_JMQC01000008.1	Bacteriocin	4785655	4795915	NA	NA	NA
9.1	NZ_JMQC01000009.1	NRPS	1	45019	NA	NA	NA
9.2	NZ_JMQC01000009.1	NRPS	193574	314941	Iturin	NRP + polyketide	33
11.1	NZ_JMQC01000011.1	NRPS	131166	179247	NA	NA	NA
11.2	NZ_JMQC01000011.1	NRPS	218114	262205	Puwainaphycin A/B/C/D	NRP + polyketide	30

a BGCs were identified using the bacterial version of the antiSMASH Web server (https://antismash.secondarymetabolites.org/#!/start, accessed 17 July 2020) in “relaxed” detection mode.

b NRPS, nonribosomal peptide synthetase; LAP, linear azol(in)e-containing peptide.

c NA, not available; assigned to predicted BGCs that could not be assigned to a most similar known BGC.

d MIBiG, the Minimum Information about a Biosynthetic Gene cluster database (https://mibig.secondarymetabolites.org/); RiPP, ribosomally synthesized and posttranslationally modified peptide; NRP, nonribosomal peptide.

### Unlike the B. mycoides and *B. paramycoides* type strains, ATCC 21929 is oxidase negative.

ATCC 21929 cells stained Gram positive and were approximately 3 μm long. Morphology of ATCC 21929 was observed by transmission electron microscopy (Fig. S2 and Text S1). ATCC 21929 was hemolytic and oxidase negative. The strain was able to hydrolyze starch and casein at 32°C after 72 h of incubation, indicating that it possesses both amylase and caseinase activity. ATCC 21929 was motile and grew a visible biomass after 3 days of incubation under anaerobic conditions. It grew at temperatures of 15 to 43°C, pH of 6 to 9, and NaCl concentrations of 0 to 5% (Table 3). The spore-forming capabilities of ATCC 21929 were not specifically assessed.

**TABLE 3:** Phenotypic characteristics of novel effective species “*B. clarus*” strain ATCC 21929^T^ and published and effective Bacillus cereus group species type strains^*i*^

Characteristic	Value for species type strain:																					
	1	2	3	4	5	6	7	8	9	10	11	12	13	14	15	16	17	18	19	20	21	22
Oxidase test	−	+	+	+	+	+	−	+	−	+	+	+	+	+	+	+	+	+	+	+	+	−
Temp range (°C)	15–43	15–40	10–50	15–45	10–45	20–50	15–40	10–39	15–40	10–39	15–40	7–39	15–45	15–39	15–45	10–39	10–40	10–45	15–45	10–45	5–37	5–43
NaCl range (%, wt/vol)	0–5	0–9	NR	0–4	0–4	NR	0–6	0–7	<7	0–9	0–4	0–9	0–9	0–5	0–9	0–9	0–2.5	0–5	0–9	0–4	NR	0–5
pH range	6–9	5–10	NR	4–10	5–9.5	NR	5–9	5–10	5–9	5–9	5–9.5	5–9	5–10	5–9	5–10	5–10	5–9.5	5–9.5	5–9	5–9.5	NR	5–10
Optimal temp (°C)	37	30	NR	30	30 (37)	30–37	30	30	NR	30	30	30	30	30	30	30	30	35	30	30	NR	20–40
Optimal pH	6–7	7	NR	7	6	NR	7	7	7	7	8	7	6	7	7–8	8	8	6.5	6	7	NR	NR
Optimal NaCl concn (%, wt/vol)	0.5–3	0.5–1	NR	0–2	0	NR	NR	0.5	NR	0	1	0	1	0.5	1–2	0–1	1	0	0–0.5	0	NR	NR
Hemolysis test	+	NR	(−)^*b*^	NR	(+)^*b*^	NR	NR	NR	NR	NR	(+)^*f*^	NR	NR	NR	NR	NR	(+)^*f*^	NR	NR	(+)^*c*^	(+)^*f*^	NR
Casein hydrolysis	+	+	NR	NR	(+)^*d*^	NR	(−)^*d*^	+	(−)^*d*^	+	(+)^*d*^	+	+	+	+	+	(+)^*d*^	NR	+	(+)^*d*^	(+)^*d*^	(+)^*a*^
Motility	+	−	(−)^*a*^	(+)^*e*^	(+)^*a*^	(+)^*a*^	NR	+	(+)^*d*^	+	(−)^*g*^	−	−	−	−	−	(+)^*a*^	(+)^*a*^	+	(+)^*a*^	(+)^*a*^	(+)^*a*^
Anaerobic growth	+	+	(+)^*a*^	NR	(+)^*a*^	(+w)^*a*^	(+)^*d*^	+	(+)^*d*^	+	(+)^*a*^	+	+	+	+	+	(+)^*a*^	(+)^*a*^	+	(+)^*a*^	(+)^*h*^	(+)^*a*^
Starch hydrolysis	+	+	+	+	+	−	−	−	−	+	+	−	−	+	−	+	−	+	+	+	+	+
Catalase test	+	+	+	+	+	+	+	+	+	+	+	+	+	+	+	+	+	+	+	+	+	+
Tryptophan deaminase	+	−	−	−	−	−	−	−	−	−	−	−	−	−	−	−	−	−	−	−	−	−
β-Galactosidase	−	−	−	−	−	−	−	−	−	+	−	−	−	−	−	−	−	−	−	−	−	−
Arginine dihydrolase	+	+	+	−	+	−	+	−	−	−	−	+	+	−	+	+	+	+	+	+	+	−
Citrate utilization	−	+	−	+	+	−	+	+	−	+	−	+	+	+	+	+	−	+	+	+	−	+
Urease	+	−	−	−	−	−	+	−	−	−	−	−	−	−	−	−	−	−	−	−	−	−
Voges-Proskauer reaction	+	+	+	−	+	+w	+	+	−	+	+	+	+	+	+	+	+	+	+	+	+	+
Gelatinase	+	+	v	−	+	+	+	+	+	+	+	+	+	+	+	+	+	+	+	+	+	+
Fermentation/oxidation (glucose)	−	−	−	−	−	−	−	−	−	−	−	−	−	+	−	−	−	−	−	−	−	−
Glycerol	+w	−	−	+	−	−	−	−	−	−	−	−	−	−	−	−	+(−w)	−	+	−	+w	−
d-Ribose	+	+	+	+	+	+	+	+	+	+	+w	+	+	+	+	+	+	+	+	−(+w)	+w	+
d-Xylose	−	−	−	−	−	−	−	−	−	−	−	−	+	−	−	−	−	−	−	−	−	−
d-Galactose	−	−	−	−	−	−	−	−	−	−	−	−	−	+	−	−	−	−	−	−	−	−
d-Glucose	+	+	+	+	+	+	+	+	+	+	+	+	+	+	+	+	+	+	−	+	+	+
d-Fructose	+	+	+	+	+	+	+	+	−	−	+	+	+	+	+	+	+	+	+	+	+	+
d-Mannose	−	−	−	−	−	+	−	−	−	−	−	−	−	+	−	−	−	−	NR	+	−	−
Methyl-α-d-glucopyranoside	−	−	−	−	−	−	−	−	−	−	−	−	−	−	−	−	−	+	−	−	−	−
*N-*Acetylglucosamine	+	+	+	+	+	+	−	+	+	−	+	+	+	+	+	+	+	+	−	+	+	+
Amygdalin	+w	−	−	−	+w	+w	−	−	−	−	−	−	−	+	−	−	−	+w	−	−	+w	−
Arbutin	+	+	v	−	+	+	−	+	−	+	+	+	+	+	−	+	+	+	−	+	+	+
Esculin ferric citrate	+	+	+	−	+	+	+	+	+	+	+	+	+	+	+	+	+	+	+	+	+	+
Salicin	+	+	−	+	+	+	−	+	−	+	+	+	−	+	−	+	+	+	+	+	+	+
Cellobiose	+w	+	−	+	+	+	−	+	−	−	+w	−	−	+	−	+	−	−	+	+	+w	+
Sucrose	+	+	+	+	+	−	+	+	−	−	+	−	+	−	+	−	−	+	−	+	−	−
Trehalose	−	+	+	+	+	−	+	+	−	−	+	+	+	+	+	+	+	+	+	+	+	+
Starch	−	+	+	−	+	−	+	−	−	+	+	+	−	+	−	+	+	+	+	+	+	+
Turanose	−	−	−	+	−	−	−	−	−	−	−	−	−	−	−	−	−	+	−	−	−	−
Potassium gluconate	+w	−	−	−	−	−	−	−	−	−	−	−	−	−	−	−	−	−	+	−	−	−
Maltose	+	+	+	+	+	+	+	+	+	+	+	+	+	+	+	+	+	+	+	+	+	+
DNA G+C content	35.2	35	35.2	35.5	35.3	35.9	36.6	35.5	37.1	35.3	35.2	35.3	35.2	35.2	35.2	35.2	35.4	35.6	35.2	34.8	35.2	35.2
Glycogen	+	+	+	+	+	−	+	−	−	+	+	+	−	+	−	+	+	+	+	+	+	+

a Data obtained from work of Miller et al., 2016 (8).

b Data obtained from work of Klee et al., 2006 (22).

c Data obtained from work of Ezzell et al., 1990 (23).

d Data obtained from work of Jung et al., 2011 (11).

e Data obtained from work of Liu et al., 2014 (9).

f Data obtained from work of Pruss et al., 1999 (24).

g Data obtained from work of Bergey et al., 2009 (25).

h Data obtained from work of Lechner et al., 1998 (7).

i Species numbers: 1, novel effective species “*B. clarus*” strain ATCC 21929^T^; 2, *B. albus* N35-10-2^T^; 3, B. anthracis ATCC 14578^T^; 4, “*B. bingmayongensis*” FJAT-13831^T^; 5, B. cereus sensu stricto ATCC 14579^T^; 6, *B. cytotoxicus* NVH 391-98^T^; 7, “*B. gaemokensis*” BL3-6^T^; 8, *B. luti* TD41^T^; 9, “*B. manliponensis*” BL4-6^T^; 10, *B. mobilis* 0711P9-1^T^; 11, B. mycoides DSM 2048^T^; 12, *B. nitratireducens* 4049^T^; 13, *B. pacificus* EB422^T^; 14, *B. paramycoides* NH24A2^T^; 15, *B. paranthracis* Mn5^T^; 16, *B. proteolyticus* TD42^T^; 17, B. pseudomycoides DSM 12442^T^; 18, *B. toyonensis* BCT-7112^T^; 19, *B. tropicus* N24^T^; 20, B. thuringiensis ATCC 10792^T^; 21, B. weihenstephanensis DSM 11821^T^; 22, *B. wiedmannii* FSL W8-0169^T^. The data for “*B. clarus*” strain ATCC 21929^T^ were produced in this study. All other data were obtained from the work of Liu et al., 2017 (1), unless specified otherwise in the footnotes. In the API 20E tests, all strains were negative for lysine decarboxylase, ornithine decarboxylase, H_2_S production, indole production, mannitol, inositol, sorbitol, rhamnose, melibiose, and arabinose. In the API 50 CHB tests, all strains were positive for maltose and negative for erythritol, d-arabinose, l-arabinose, l-xylose, d-adonitol, methyl-β-d-xylopyranoside, l-sorbose, l-rhamnose, dulcitol, inositol, d-mannitol, d-sorbitol, methyl-α-d-mannopyranoside, lactose, melibiose, inulin, melezitose, raffinose, xylitol, gentiobiose, d-lyxose, d-tagatose, d-fucose, l-fucose, d-arabitol, l-arabitol, potassium 2-ketogluconate, and potassium 5-ketogluconate. −, negative; −w, weakly negative; +, positive; +w, weakly positive; v, variable; NR, not reported. In cases where a second phenotype was reported, the secondary phenotype is listed in parentheses.

10.1128/mSphere.00882-20.3 FIG S2 Transmission electron microscopy image of novel effective B. cereus group species “*B. clarus*” strain ATCC 21929^T^. Image was obtained using 2% uranyl acetate staining. Download FIG S2, PDF file, 0.8 MB. Copyright © 2020 Méndez Acevedo et al. 2020 Méndez Acevedo et al. This content is distributed under the terms of the Creative Commons Attribution 4.0 International license.

Fatty acid composition of ATCC 21929 (Text S1) revealed that iso-C15:0 was most abundant. Among the least abundant fatty acids were C15:1 ω5c and iso-11:0 3OH. The latter two fatty acids, along with iso-13:0 3OH, were not reported for any other B. cereus group species type strain (Table 4). API 20E and CH50 biochemical assays (bioMérieux; Text S1) indicated that ATCC 21929 has a metabolic capacity similar to that of other B. cereus group species type strains (Table 4).

**TABLE 4:** Fatty acid composition of novel effective species “*B. clarus*” strain ATCC 21929^T^ and other published and effective Bacillus cereus group species type strains^*a*^

Fatty acid	Value for species type strain:																				
	1	2	4	5	6	7	8	9	10	11	12	13	14	15	16	17	18	19	20	21	22
C_12:0_	0.67	2.7	NR	1.4	TR	2.3	1.3	5.9	1.8	2.7	4.7	1.7	1.5	1.6	3.3	1.4	NR	1	1	2.5	TR
C_14:0_	3.56	7.9	4.1	4.1	2.4	5	6.9	9	3.6	3.7	7.7	6.8	3.9	5.4	6.5	3.2	3.2	5.5	4.1	3.6	3.3
C_15:1_ ω5c	0.2	NR	NR	NR	NR	NR	NR	NR	NR	NR	NR	NR	NR	NR	NR	NR	NR	NR	NR	NR	NR
C_16:0_	5.43	16.4	9.8	12.5	10.8	12.5	14.8	21	16.7	15.6	30.5	19.9	33	14.6	33.3	9	5.6	12	10.3	18	7.3
C_16:1_ ω6c	NR	NR	NR	5.9	3.3	NR	NR	NR	NR	6.4	NR	NR	NR	NR	NR	12.3	NR	NR	7.5	3.6	NR
C_16:1_ ω11c	NR	1.2	NR	TR	NR	NR	1.6	NR	1.3	1.2	2.4	1.7	1.2	1	2.1	NR	NR	1	TR	1.1	1.1
C_16:1_ ω7c alcohol	NR	TR	NR	NR	NR	NR	TR	NR	1.7	NR	TR	TR	TR	TR	TR	NR	NR	1.3	NR	NR	1.9
C_18:0_	0.32	6.1	1.7	TR	NR	2.7	2.6	5	3.4	1.6	9.2	2	5.5	1.8	5.3	TR	NR	TR	TR	1.3	TR
C_18:1_ ω9c	NR	2	NR	TR	TR	1.4	2	1.7	3	TR	1.6	3	TR	2.4	1	TR	TR	TR	TR	TR	NR
Iso-C_11:0_	0.3	NR	NR	NR	NR	NR	NR	NR	NR	NR	NR	NR	NR	NR	NR	NR	NR	NR	NR	NR	NR
Iso-C_12:0_	0.66	1.3	TR	1.9	TR	3	1.1	4.9	1.9	2.6	2.2	1.2	1.5	1.4	2.3	8.7	NR	TR	1.5	2.9	TR
Iso-C_13:0_	14.84	6.9	7.7	20.3	7	7.9	6.8	5	4.9	21.9	8.5	6.4	7.9	7	8.8	12.6	7.1	7.1	18.5	22.3	6.9
Iso-C_14:0_	2.3	4.2	2.9	4.8	5	5.9	4	6.3	5.4	3.4	1.8	3.5	1.9	5.5	2.7	5.5	2.3	5.9	5.2	3.5	5.1
Iso-C_15:0_	32.02	1	21	20.2	36.5	10.6	14.9	4	10.8	12.5	5.5	13.8	9	14.7	8.3	13.3	38.6	18.9	21.8	12.6	27.6
Iso-C_15:1_ G	NR	1.2	NR	NR	NR	TR	TR	TR	TR	NR	TR	TR	TR	NR	TR	NR	NR	TR	NR	NR	NR
Iso-C_16:0_	2.59	4.6	3.6	3.3	6.7	5.3	5.6	5.7	9.1	2.8	3.2	5.3	3.6	7.4	3.4	8.3	5.1	7.8	3.7	2.9	9.1
Iso-C_16:1_ ω-5	NR	NR	NR	TR	TR	NR	NR	NR	NR	TR	NR	NR	NR	NR	NR	2.9	NR	NR	1.3	TR	NR
Iso-C_17:0_	12.15	3.8	11.5	6.7	8.2	5.3	6.6	2.7	6	7.5	2.5	5.2	8.5	6.5	4.3	7	11.4	9.6	6.9	6.6	10.1
Iso-C_17:1_ ω-11	NR	NR	NR	2.7	TR	NR	NR	NR	NR	6.5	NR	NR	NR	NR	NR	TR	NR	NR	3.2	4.2	NR
Iso-C_17:1_ ω-6	NR	NR	NR	1	TR	NR	NR	NR	NR	1	NR	NR	NR	NR	NR	2.3	NR	NR	2.6	TR	NR
Iso-C_17:1_ ω10c	NR	TR	NR	NR	NR	NR	1.8	NR	2.4	NR	1.8	1.7	1.1	1.7	1.6	NR	5.8	3.5	NR	NR	4.7
Iso-_17:1_ ω5c	5.8	1.3	5.1	NR	NR	2.6	2.7	NR	NR	NR	TR	1	NR	2.1	TR	NR	4.9	2.6	NR	NR	2.6
Anteiso-C_13:0_	0.98	1.3	2.2	4	1.8	3.5	1.4	3.3	1.8	3.9	2.4	2	2.5	1.7	2.9	4.9	NR	1	2.8	5.8	1
Anteiso-C_15:0_	2.16	4.4	7.4	6.5	10.8	5.5	4.6	3.3	6.5	3.8	2.4	6.7	4.2	5.6	3.9	3.6	3.1	4.4	5.3	5.4	4
Anteiso-C_17:0_	0.6	1.6	2.8	1.5	3.4	2	1.8	1.5	3.2	1.1	1.1	2.7	2	2.2	1.5	1.6	NR	1.7	1.1	1.7	1.5
Anteiso-C_17:1_ a	0.55	TR	TR	NR	NR	1.3	TR	TR	1	NR	TR	TR	TR	1	NR	NR	NR	TR	NR	NR	TR
Anteiso-C_17:1_ ω-6	NR	NR	NR	TR	TR	NR	NR	NR	NR	TR	NR	NR	NR	NR	NR	1.1	NR	NR	TR	TR	NR
Iso-11:0 3OH	0.22	NR	NR	NR	NR	NR	NR	NR	NR	NR	NR	NR	NR	NR	NR	NR	NR	NR	NR	NR	NR
Iso-13:0 3OH	0.46	NR	NR	NR	NR	NR	NR	NR	NR	NR	NR	NR	NR	NR	NR	NR	NR	NR	NR	NR	NR

a Species: 1, novel effective species “*B. clarus*” strain ATCC 21929^T^; 2, *B. albus* N35-10-2^T^; 3, B. anthracis ATCC 14578^T^ (no data to show); 4, “*B*. *bingmayongensis*” FJAT-13831^T^; 5, B. cereus sensu stricto ATCC 14579^T^; 6, *B. cytotoxicus* NVH 391-98^T^; 7, “*B. gaemokensis*” BL3-6^T^; 8, *B. luti* TD41^T^; 9, “*B. manliponensis*” BL4-6^T^; 10, *B. mobilis* 0711P9-1^T^; 11, B. mycoides DSM 2048^T^; 12, *B. nitratireducens* 4049^T^; 13, *B. pacificus* EB422^T^; 14, *B. paramycoides* NH24A2^T^; 15, *B. paranthracis* Mn5^T^; 16, *B. proteolyticus* TD42^T^; 17, B. pseudomycoides DSM 12442^T^; 18, *B. toyonensis* BCT-7112^T^; 19, *B. tropicus* N24^T^; 20, B. thuringiensis ATCC 10792^T^; 21, B. weihenstephanensis DSM 11821^T^; 22, *B. wiedmannii* FSL W8-0169^T^. The data for “*B. clarus*” strain ATCC 21929^T^ were produced in this study. All other data were obtained from the work of Liu et al., 2017 (1). NR, not reported; TR, trace amount.

### “*B. clarus*” is an effective B. cereus group species.

By all contemporary metrics used to delineate prokaryotic species, singleton genome ATCC 21929 represents a novel B. cereus group species. However, in order to validly publish a novel species, its type strain cannot be patented, as is the case here (13, 18). Furthermore, ATCC 21929 had been deposited in the American Type Culture Collection (ATCC), which did not allow for its deposition in another international culture collection (a hard requirement for new species validation) (18). ATCC 21929 should be evaluated as a member of a novel effective species, for which we propose the name “*B. clarus*.”

### Description of effective species “*Bacillus clarus*.”

“*Bacillus clarus*” (cla′rus. L. masc. adj. *clarus* clear).

Cells stained Gram positive and displayed a long rod-like appearance, 3 μm in length. “*B. clarus*” ATCC 21929^T^ is highly motile, oxidase negative, hemolytic, possesses amylase and caseinase activity, can reach stationary phase in 16 h when grown at 32°C in brain heart infusion (BHI), and can grow under aerobic and anaerobic conditions. “*B. clarus*” ATCC 21929^T^ can grow at pH 6 to 9, temperatures of 15 to 43°C, and NaCl concentrations of 0 to 5%; optimal conditions for growth are 6 to 9, 37°C, and 0.5 to 3%, respectively. “*B. clarus*” ATCC 21929^T^ shows weak Hbl production at 32°C, as indicated by faint bands in the Duopath Enterotoxins test, but does not reduce the metabolic activity of HeLa cells under the tested conditions. The most abundant fatty acid was iso-C15:0. Among the least abundant fatty acids were C15:1 ω5c and iso-11:0 3OH. The latter two fatty acids, along with iso-13:0 3OH, were not reported for any other B. cereus group type strain. Unique characteristics of “*B. clarus*” ATCC 21929^T^ include a higher abundance of iso-C17:0, lower abundance of iso-C16:0, and the ability to grow optimally at 3% NaCl. “*B. clarus*” ATCC 21929^T^ is oxidase negative, a trait shared only by B. cereus group members B. wiedmannii, “B. gaemokensis,” and “B. manliponensis.”

### Availability of data.

The “*B. clarus*” ATCC 21929 genome is available under NCBI RefSeq accession no. GCF_000746925.1 (original genome sequenced by Los Alamos National Laboratory) (14) and NCBI accession no. QVOD00000000 (the resequencing effort described here). “*B. clarus*” ATCC 21929 has been deposited in the PubMLST Isolates database under ID number 2468.

10.1128/mSphere.00882-20.4 FIG S3 Clusters of Orthologous Groups (COG) functional categories assigned to functionally annotated protein-encoding sequences in the genome of novel effective B. cereus group species “*B. clarus*” strain ATCC 21929^T^ (*n *= 4,876 out of 5,328 total protein-encoding sequences). Functional annotation of the ATCC 21929^T^ genome was performed via the eggNOG-mapper Web server (http://eggnog-mapper.embl.de/, accessed 20 July 2020), using the RefSeq protein sequences of ATCC 21929^T^ as input. The percentage (%) of functionally annotated protein-encoding genes assigned to a particular COG or COG group is displayed on the *y* axis. COG abbreviations are displayed on the *x* axis and correspond to the following: (NA), not assigned (no COG was assigned to the gene); B, chromatin structure and dynamics; C, energy production and conversion; D, cell cycle control, cell division, chromosome partitioning; E, amino acid transport and metabolism; F, nucleotide transport and metabolism; G, carbohydrate transport and metabolism; H, coenzyme transport and metabolism; I, lipid transport and metabolism; J, translation, ribosomal structure and biogenesis; K, transcription; L, replication, recombination, and repair; M, cell wall/membrane/envelope biogenesis; N, cell motility; O, posttranslational modification, protein turnover, chaperones; P, inorganic ion transport and metabolism; Q, secondary metabolite biosynthesis, transport, and catabolism; S, function unknown; T, signal transduction mechanisms; U, intracellular trafficking, secretion, and vesicular transport; V, defense mechanisms; W, extracellular structures. Download FIG S3, PDF file, 0.1 MB. Copyright © 2020 Méndez Acevedo et al. 2020 Méndez Acevedo et al. This content is distributed under the terms of the Creative Commons Attribution 4.0 International license.
