# Supplementary material for: Novel Effective Bacillus cereus Group Species “Bacillus clarus” Is Represented by Antibiotic-Producing Strain ATCC 21929 Isolated from Soil
Source: mSphere. 2020 Nov 4;5(6):e00882-20. doi: 10.1128/mSphere.00882-20 (PMC7643830; doi:10.1128/mSphere.00882-20)
Supplement: TEXT S1 [file mSphere.00882-20-s0001.pdf]

Supplemental Text

Novel effective *Bacillus cereus* group species "*B. clarus*", represented by antibiotic-producing strain ATCC 21929 isolated from soil

Marysabel Méndez Acevedo[a, b], Laura M. Carroll[c], Manjari Mukherjee[a], Emma Mills[a], Lingzi Xiaoli[a], Edward G. Dudley[a], Jasna Kovac[a]#

[a] Department of Food Science, The Pennsylvania State University, University Park PA 16802, USA

[b] Department of Natural Sciences, University of Puerto Rico in Aguadilla, Calle Belt Base Ramey, P.O. Box 6150 Aguadilla 00604-6150, Puerto Rico

[c] Department of Food Science, Cornell University, Ithaca NY 14853, USA

# Corresponding Author: Jasna Kovac, 437 Rodney A. Erickson Food Science Building, University Park, PA, 16802, Phone: +1 814 865 2883, jzk303@psu.edu

Supplemental Text

Isolate acquisition and re-sequencing. The genome of strain *B. mycoides* Flugge ATCC 21929^T (RefSeq Accession GCF_000746925.1, deposited by Los Alamos National Laboratory in 2014) (1) has previously been shown to be distantly related to all published *B. cereus* group genomes and species type strains, including the type strain of *B. mycoides* DSM 2048^T (2). To further characterize this strain, a culture stock was obtained from the American Type Culture Collection (ATCC) and re-sequenced to confirm its identity. The strain was re-sequenced at the Penn State Department of Food Science as part of the FDA GenomeTrakr effort, following a previously reported procedure (3, 4). Briefly, DNA was extracted using the E.Z.N.A. bacterial DNA kit (Omega) and quantified using Qubit. Extracted DNA was used for Nextera XT library preparation following the manufacturer's protocol. The resulting library was sequenced on an Illumina MiSeq platform using a 500-cycle kit and 2×250 bp paired-end reads.

Trimmomatic version 0.36 (5) was used to trim reads and remove sequencing adapters (using default settings for paired-end reads), and FastQC version 0.11.5 (6) was used to assess the quality of the resulting trimmed reads. Contigs and scaffolds were assembled from the resulting trimmed paired-end reads using SPAdes version 3.11.1 (7), using the "careful" option and *k*-mer sizes of 99 and 127. Average coverage was calculated by mapping trimmed paired-end reads back to the resulting contigs using Samtools version 1.6 (8) and BWA MEM version 0.7.13 (9, 10), yielding a value of 122.0x. The same approach was used to calculate average coverage relative to the original ATCC 21929^T genome (sequenced by Los Alamos National Laboratory; NCBI RefSeq Assembly Accession GCF_000746925.1) (1), yielding a value of 127.6x. JSpeciesWS (accessed October 12, 2020) was used to calculate average nucleotide identity BLAST (ANIb) values between the scaffolded genome sequenced here and the original

ATCC 21929^T contigs (11), yielding pairwise ANIb values of 99.97 and 99.98, with nucleotide alignment values of 97.53 and 98.84%, respectively.

The genomic data produced here was deposited in NCBI under accession number QVOD00000000. The ATCC 21929^T genome sequenced here (NCBI RefSeq Assembly Accession GCF_003428195.1) (i) was 5,782,989 bp in length (excluding gaps), (ii) comprised 229 contigs, (iii) had a contig N50 of 95,779 bp, and (iv) a GC content of 35.2%. The original genome sequenced by Los Alamos National Laboratory (NCBI RefSeq Assembly Accession GCF_000746925.1), which (i) was 5,875,917 bp in length, (ii) comprised 12 contigs, (iii) had a contig N50 of 5,068,716 bp, and (iv) a GC content of 35.3%, was downloaded and used as the ATCC 21929^T representative genome in all further genomic analyses reported in this paper (1, 12).

16S rDNA phylogeny construction. To confirm that ATCC 21929^T was a member of the *B. cereus* group, the 16S rDNA sequence of ATCC 21929^T was extracted from its genome by aligning the ATCC 21929^T genome to a database of 16S rDNA sequences of other *B. cereus* group species type strains (Table 1) using nucleotide BLAST (blastn) version 2.5.0 (13) integrated into BTyper version 2.3.2 (14). MUSCLE version 3.8.1551 (15, 16) was used to construct an alignment of all 23 16S rDNA genes. IQ-TREE version 1.5.4 (17) was used to construct a maximum likelihood (ML) phylogeny using the 16S rDNA gene alignment as input, the optimal nucleotide substitution model (i.e., the model with the lowest Bayesian Information Criterion [BIC] value) selected using ModelFinder (i.e., the HKY+I model) (18, 19), and 1,000 replicates of the ultrafast bootstrap approximation (20). FigTree version 1.4.3 (http://tree.bio.ed.ac.uk/software/figtree/) (21) was used to annotate the resulting phylogeny (Supplemental Figure S1).

Whole-genome phylogeny construction. Amino acid sequences derived from the following genomes were downloaded and used as input for OrthoFinder v. 2.3.12 (22, 23): (i) strain ATCC 21929^{T}, (ii) the type strain/representative genomes of the 19 published *B. cereus* group species, (iii) the type strains of the three effective *B. cereus* group species, and (iv) *Bacillus panaciterrae* str. DSM 19096^{T} (NCBI Assembly Accession GCF_000430785.1), the type strain of *B. panaciterrae* (24) (*B. panaciterrae* str. DSM 19096^{T} would be treated as an outgroup for the *B. cereus* group whole-genome phylogeny, as it itself is not a member of the *B. cereus* group but shares >70 ANI with each of the "*B. manliponensis*", *B. cytotoxicus, B. cereus s.s. B. anthracis, B. pseudomycoides, B. mycoides, B. toyonensis,* and *B. wiedmannii* type strain/species representative genomes [calculated using JSpeciesWS, accessed August 19, 2020]; Table 1). OrthoFinder's configuration of MAFFT v. 7.470 (25, 26) was used to construct sequence alignments, and the resulting species tree alignment (SpeciesTreeAlignment.fa) was used to construct a ML phylogeny using IQ-TREE version 1.5.4, the optimal protein substitution model selected using ModelFinder (i.e., the JTT+F+R5 model) (27-29), and 1,000 replicates of the ultrafast bootstrap approximation. FigTree version 1.4.3 was used to annotate the phylogeny (Figure 1).

Calculation of average nucleotide identity and *in silico* DNA-DNA hybridization values. FastANI version 1.0 (30) and JSpeciesWS (http://jspecies.ribohost.com/jspeciesws/; accessed July 15, 2020) (11) were used to calculate average nucleotide identity (ANI) values between the ATCC 21929^{T} genome and type strain/species representative genomes of each of the 19 published and three effective *B. cereus* group species (Table 1 and Supplemental Table S1). The Genome-to-Genome Distance Calculator (GGDC; https://ggdc.dsmz.de/, accessed July 15, 2020)

(31) was used to calculate *in silico* DNA-DNA hybridization (DDH) values between ATCC 21929^T and the set of 22 genomes (Table 1 and Supplemental Table S1).

***In silico* single- and multi-locus sequence typing and virulence gene detection.** BTyper version 2.3.2 was additionally used to perform the following *in silico* analyses, using each of 23 *B. cereus* group genomes as input (Table 1): (i) virulence gene detection, using default minimum amino acid identity and coverage thresholds of 50 and 70%, respectively; (ii) *panC* group assignment, using the seven-group framework described by Guinebretiere, et al. (32, 33); (iii) seven-gene multi-locus sequence typing (MLST), using the PubMLST MLST scheme for *B. cereus* (34, 35); (iv) *rpoB* allelic typing using the Cornell University Food Safety Laboratory and Milk Quality Improvement Program (CUFSL/MQIP) *rpoB* allelic typing scheme (36) (Table 1 and Supplemental Table S1). The Sym'Previus *B. cereus* group *panC* group assignment web server (https://tools.symprevius.org/Bcereus/; accessed July 16, 2020) (33) and BTyper3 version 3.1.0 (2) were additionally used to assign each genome to a *panC* group using the seven-group scheme described by Guinebretiere, et al. (32, 33) and a recently proposed adjusted eight-group scheme (37), respectively (Table 1 and Supplemental Table S1).

Microbiological, biochemical, and phenotypic characterization. All phenotypic tests were conducted using *B. cereus sensu stricto* (*s.s.*) strain ATCC 14579^T as a control strain. ATCC 21929^T was examined microscopically using a Gram stain and a light microscope, as well as a 2% uranyl acetate negative stain and a transmission electron microscope (Supplemental Figure S2).

The ability of ATCC 21929^T to produce diarrheal enterotoxins hemolysin BL (Hbl) and non-hemolytic enterotoxin (Nhe) was assessed using a Duopath Cereus Enterotoxins kit (Merck). The culture was grown to stationary phase in BHI at 32°C and at 37°C, without shaking. The

culture was then applied to the lateral flow device and the assay results were read following the manufacturer's instructions. *B. cereus s.s.* strain ATCC 14579^{T}, which was used as a control, was positive for production of both toxins at both temperatures.

The cytotoxic potential of strain ATCC 21929^{T} was assessed in a 96-well microtiter plate by incubating 12 replicates of confluent HeLa cells with 5% v/v bacterial supernatant (bacteria grown at 37°C) for 15 min, followed by the addition of 10 µl of WST-1 dye solution (Roche) and a further 25-min incubation (38). The final absorbance was determined by subtracting the absorbance values measured at 690 nm from those measured at 450 nm. Percent viability was determined relative to cells treated with 5% v/v BHI (negative cytotoxicity control). Cells treated with 0.05% Triton X-100 were used as a positive cytotoxicity control (38). The *B. cereus s.s.* and *B. pseudomycoides* type strains exhibited strong cytotoxic effects, consistent with previous reports (Figure 2) (38, 39).

ATCC 21929^{T} was further characterized phenotypically by following protocols outlined in Bergey's manual (40). Briefly, a loopful of 24-hour culture suspension was streaked onto blood agar, followed by incubation at 35°C for 24 h to examine the strain's hemolytic activity. The culture was also tested for oxidase activity using Oxidrop reagent (Hardy Diagnostics), and for amylase and caseinase activity by growing on starch and casein agars, respectively, at 32°C after 72 h. The ability of the strain to grow in anaerobic conditions was tested by inoculating anaerobic agar with an overnight culture and incubating it in a jar with an anaerobic gas pack at 30°C for 7 days. Motility was examined by preparing a Motility Test Medium according to the Bacteriological Analytical Manual (BAM) (41), stab-inoculating overnight culture, and incubating it for 24 h at 32°C. The strain's ability to grow at different temperatures (4, 7, 10, 15, 20, 25, 30, 37, 40, 43, 45, and 55°C) was assessed by streaking individual well-isolated colonies

on BHI agar plates, in triplicate, and incubating them up to 3 weeks or until growth was observed (40). The ability of ATCC 21929^T to grow at different pH was confirmed by inoculating 10 µl of an overnight culture into BHI broths adjusted to pH 3-11 using appropriate buffers, in triplicate. Citrate buffer was used to supplement BHI adjusted to pH 3, 4, and 5, phosphate buffer was added to BHI adjusted to pH 6, 7, and 8, and CAPS buffer was added to BHI adjusted to pH 9, 10, and 11. Inoculated BHI tubes were incubated at 30°C for 14 days or until growth was observed based on turbidity. The strain's ability to grow at different concentrations of NaCl was determined by supplementing TSB broth with 0, 0.5, 1, 2, 3, 5, 7, 9, 12, and 15% of NaCl. Tubes were inoculated with 10 µl of an overnight culture, in triplicate, and incubated at 32°C for 14 days or until growth was confirmed based on turbidity. The fatty acid composition was determined by MID Inc. for culture grown at their standard conditions, on tryptic soy agar at 28°C. API 20E and CH50 biochemical assays (bioMérieux) were performed following the manufacturer's instructions at 32°C. The spore-forming capabilities of ATCC 21929 were not specifically assessed.

Biosynthetic gene cluster detection and functional annotation. The bacterial version of the antiSMASH web server (https://antismash.secondarymetabolites.org/#!/start, accessed July 17, 2020) (42) was used in "relaxed" detection mode to identify potential biosynthetic gene clusters (BGCs) in the genome of ATCC 21929^T. Functional annotation of the ATCC 21929^T genome was performed via the eggNOG-mapper webserver (http://eggnog-mapper.embl.de/, accessed July 20, 2020) (43, 44), using the RefSeq protein sequences of ATCC 21929^T as input. The ggplot2 (45) package in R version 3.6.1 (46) was used to construct a bar plot of the resulting Clusters of Orthologous Groups (COG) functional categories (47) assigned to the protein sequences (Supplemental Figure S3).

Supplemental Text References

1. Daligault HE, Davenport KW, Minogue TD, Bishop-Lilly KA, Broomall SM, Bruce DC, Chain PS, Coyne SR, Frey KG, Gibbons HS, Jaissle J, Koroleva GI, Ladner JT, Lo C-C, Munk C, Palacios GF, Redden CL, Rosenzweig CN, Scholz MB, Johnson SL. 2014. Twenty Whole-Genome *Bacillus* sp. Assemblies. Genome Announcements 2:e00958-14.
2. Carroll LM, Wiedmann M, Kovac J. 2020. Proposal of a Taxonomic Nomenclature for the *Bacillus cereus* Group Which Reconciles Genomic Definitions of Bacterial Species with Clinical and Industrial Phenotypes. mBio 11:e00034-20.
3. Nwanosike H, Chung T, Xiaoli L, Condello M, Dudley EG, Kovac J. 2019. Whole-Genome Sequences of *Escherichia coli* Isolates from Cocoa Beans Imported from Bolivia. Microbiology Resource Announcements 8:e01516-18.
4. Connolly CJ, Kaminsky L, Pinto GN, Sinclair PC, Bajracharya G, Yan R, Nawrocki EM, Dudley EG, Kovac J. 2020. Whole-Genome Sequences of *Salmonella* Isolates from an Ecological Wastewater Treatment System. Microbiology Resource Announcements 9:e00456-20.
5. Bolger AM, Lohse M, Usadel B. 2014. Trimmomatic: a flexible trimmer for Illumina sequence data. Bioinformatics 30:2114-20.
6. Andrews S. 2019. FastQC: a quality control tool for high throughput sequence data, v0.11.8. https://www.bioinformatics.babraham.ac.uk/projects/fastqc/.
7. Bankevich A, Nurk S, Antipov D, Gurevich AA, Dvorkin M, Kulikov AS, Lesin VM, Nikolenko SI, Pham S, Prjibelski AD, Pyshkin AV, Sirotkin AV, Vyahhi N, Tesler G, Alekseyev MA, Pevzner PA. 2012. SPAdes: a new genome assembly algorithm and its applications to single-cell sequencing. J Comput Biol 19:455-77.
8. Li H, Handsaker B, Wysoker A, Fennell T, Ruan J, Homer N, Marth G, Abecasis G, Durbin R, Genome Project Data Processing S. 2009. The Sequence Alignment/Map format and SAMtools. Bioinformatics 25:2078-9.
9. Li H. 2013. Aligning sequence reads, clone sequences and assembly contigs with BWA-MEM. arXiv:1303.3997.
10. Li H, Durbin R. 2009. Fast and accurate short read alignment with Burrows-Wheeler transform. Bioinformatics 25:1754-60.
11. Richter M, Rossello-Mora R, Oliver Glockner F, Peplies J. 2016. JSpeciesWS: a web server for prokaryotic species circumscription based on pairwise genome comparison. Bioinformatics 32:929-31.
12. Pruitt KD, Tatusova T, Maglott DR. 2007. NCBI reference sequences (RefSeq): a curated non-redundant sequence database of genomes, transcripts and proteins. Nucleic Acids Res 35:D61-5.
13. Camacho C, Coulouris G, Avagyan V, Ma N, Papadopoulos J, Bealer K, Madden TL. 2009. BLAST+: architecture and applications. BMC Bioinformatics 10:421.
14. Carroll LM, Kovac J, Miller RA, Wiedmann M. 2017. Rapid, High-Throughput Identification of Anthrax-Causing and Emetic *Bacillus cereus* Group Genome Assemblies via BTyper, a Computational Tool for Virulence-Based Classification of *Bacillus cereus* Group Isolates by Using Nucleotide Sequencing Data. Appl Environ Microbiol 83.
15. Edgar RC. 2004. MUSCLE: a multiple sequence alignment method with reduced time and space complexity. BMC Bioinformatics 5:113.

16. Edgar RC. 2004. MUSCLE: multiple sequence alignment with high accuracy and high throughput. Nucleic Acids Res 32:1792-7.
17. Nguyen LT, Schmidt HA, von Haeseler A, Minh BQ. 2015. IQ-TREE: a fast and effective stochastic algorithm for estimating maximum-likelihood phylogenies. Mol Biol Evol 32:268-74.
18. Kalyaanamoorthy S, Minh BQ, Wong TKF, von Haeseler A, Jermiin LS. 2017. ModelFinder: fast model selection for accurate phylogenetic estimates. Nat Methods 14:587-589.
19. Hasegawa M, Kishino H, Yano T. 1985. Dating of the human-ape splitting by a molecular clock of mitochondrial DNA. J Mol Evol 22:160-74.
20. Minh BQ, Nguyen MA, von Haeseler A. 2013. Ultrafast approximation for phylogenetic bootstrap. Mol Biol Evol 30:1188-95.
21. Rambaut A. 2016. FigTree: a graphical viewer of phylogenetic trees, v1.4.3. http://tree.bio.ed.ac.uk/software/figtree/.
22. Emms DM, Kelly S. 2019. OrthoFinder: phylogenetic orthology inference for comparative genomics. Genome Biol 20:238.
23. Emms DM, Kelly S. 2015. OrthoFinder: solving fundamental biases in whole genome comparisons dramatically improves orthogroup inference accuracy. Genome Biol 16:157.
24. Ten LN, Baek SH, Im WT, Liu QM, Aslam Z, Lee ST. 2006. *Bacillus panaciterrae* sp. nov., isolated from soil of a ginseng field. Int J Syst Evol Microbiol 56:2861-2866.
25. Katoh K, Misawa K, Kuma K, Miyata T. 2002. MAFFT: a novel method for rapid multiple sequence alignment based on fast Fourier transform. Nucleic Acids Res 30:3059-66.
26. Katoh K, Standley DM. 2013. MAFFT multiple sequence alignment software version 7: improvements in performance and usability. Mol Biol Evol 30:772-80.
27. Jones DT, Taylor WR, Thornton JM. 1992. The rapid generation of mutation data matrices from protein sequences. Bioinformatics 8:275-282.
28. Yang Z. 1995. A space-time process model for the evolution of DNA sequences. Genetics 139:993-1005.
29. Soubrier J, Steel M, Lee MSY, Der Sarkissian C, Guindon S, Ho SYW, Cooper A. 2012. The Influence of Rate Heterogeneity among Sites on the Time Dependence of Molecular Rates. Molecular Biology and Evolution 29:3345-3358.
30. Jain C, Rodriguez RL, Phillippy AM, Konstantinidis KT, Aluru S. 2018. High throughput ANI analysis of 90K prokaryotic genomes reveals clear species boundaries. Nat Commun 9:5114.
31. Meier-Kolthoff JP, Auch AF, Klenk H-P, Göker M. 2013. Genome sequence-based species delimitation with confidence intervals and improved distance functions. BMC Bioinformatics 14:60.
32. Guinebretiere MH, Thompson FL, Sorokin A, Normand P, Dawyndt P, Ehling-Schulz M, Svensson B, Sanchis V, Nguyen-The C, Heyndrickx M, De Vos P. 2008. Ecological diversification in the *Bacillus cereus* Group. Environ Microbiol 10:851-65.
33. Guinebretiere MH, Velge P, Couvert O, Carlin F, Debuyser ML, Nguyen-The C. 2010. Ability of *Bacillus cereus* group strains to cause food poisoning varies according to phylogenetic affiliation (groups I to VII) rather than species affiliation. J Clin Microbiol 48:3388-91.

34. Jolley KA, Maiden MC. 2010. BIGSdb: Scalable analysis of bacterial genome variation at the population level. BMC Bioinformatics 11:595.
35. Jolley KA, Bray JE, Maiden MCJ. 2018. Open-access bacterial population genomics: BIGSdb software, the PubMLST.org website and their applications. Wellcome Open Res 3:124.
36. Ivy RA, Ranieri ML, Martin NH, den Bakker HC, Xavier BM, Wiedmann M, Boor KJ. 2012. Identification and characterization of psychrotolerant sporeformers associated with fluid milk production and processing. Appl Environ Microbiol 78:1853-64.
37. Carroll LM, Cheng RA, Kovac J. 2020. No Assembly Required: Using BTyper3 to Assess the Congruency of a Proposed Taxonomic Framework for the *Bacillus cereus* Group With Historical Typing Methods. Frontiers in Microbiology 11.
38. Carroll LM, Wiedmann M, Mukherjee M, Nicholas DC, Mingle LA, Dumas NB, Cole JA, Kovac J. 2019. Characterization of Emetic and Diarrheal *Bacillus cereus* Strains From a 2016 Foodborne Outbreak Using Whole-Genome Sequencing: Addressing the Microbiological, Epidemiological, and Bioinformatic Challenges. Front Microbiol 10:144.
39. Miller RA, Jian J, Beno SM, Wiedmann M, Kovac J. 2018. Intraclade Variability in Toxin Production and Cytotoxicity of *Bacillus cereus* Group Type Strains and Dairy-Associated Isolates. Appl Environ Microbiol 84.
40. Bergey DH, Whitman WB, De Vos P, Garrity GM, Jones D. 2009. Bergey's manual of systematic bacteriology. Vol. 3, The firmicutes, vol 3. Springer, New York.
41. Tallent SM, Knolhoff A, Rhodehamel EJ, Harmon SM, Bennett RW. 2019. Chapter 14: *Bacillus cereus*, Bacteriological Analytical Manual (BAM), 8th ed. Food and Drug Administration.
42. Blin K, Shaw S, Steinke K, Villebro R, Ziemert N, Lee SY, Medema MH, Weber T. 2019. antiSMASH 5.0: updates to the secondary metabolite genome mining pipeline. Nucleic Acids Res 47:W81-W87.
43. Huerta-Cepas J, Forslund K, Coelho LP, Szklarczyk D, Jensen LJ, von Mering C, Bork P. 2017. Fast Genome-Wide Functional Annotation through Orthology Assignment by eggNOG-Mapper. Mol Biol Evol 34:2115-2122.
44. Huerta-Cepas J, Szklarczyk D, Heller D, Hernandez-Plaza A, Forslund SK, Cook H, Mende DR, Letunic I, Rattei T, Jensen LJ, von Mering C, Bork P. 2019. eggNOG 5.0: a hierarchical, functionally and phylogenetically annotated orthology resource based on 5090 organisms and 2502 viruses. Nucleic Acids Res 47:D309-D314.
45. Wickham H. 2016. ggplot2: Elegant Graphics for Data Analysis. Springer-Verlag New York.
46. R Core Team. 2019. R: A Language and Environment for Statistical Computing, v3.6.1. R Foundation for Statistical Computing, Vienna, Austria. https://www.R-project.org/.
47. Galperin MY, Makarova KS, Wolf YI, Koonin EV. 2014. Expanded microbial genome coverage and improved protein family annotation in the COG database. Nucleic Acids Research 43:D261-D269.
