# Supplementary figures and images for: Novel Effective Bacillus cereus Group Species “Bacillus clarus” Is Represented by Antibiotic-Producing Strain ATCC 21929 Isolated from Soil
Source: mSphere. 2020 Nov 4;5(6):e00882-20. doi: 10.1128/mSphere.00882-20 (PMC7643830; doi:10.1128/mSphere.00882-20)

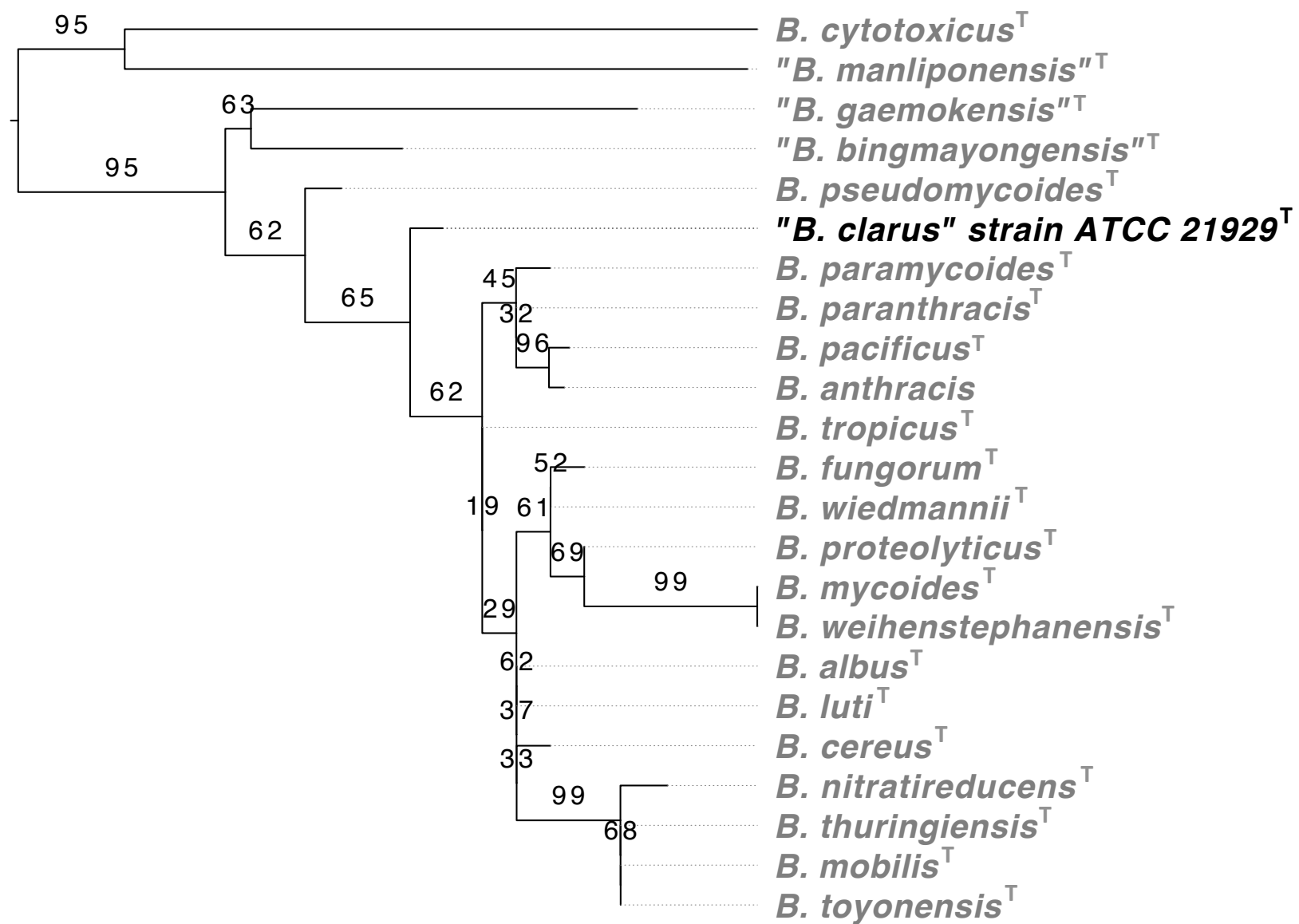

0.002

Supplement: FIG S1 [file mSphere.00882-20-sf001.pdf]

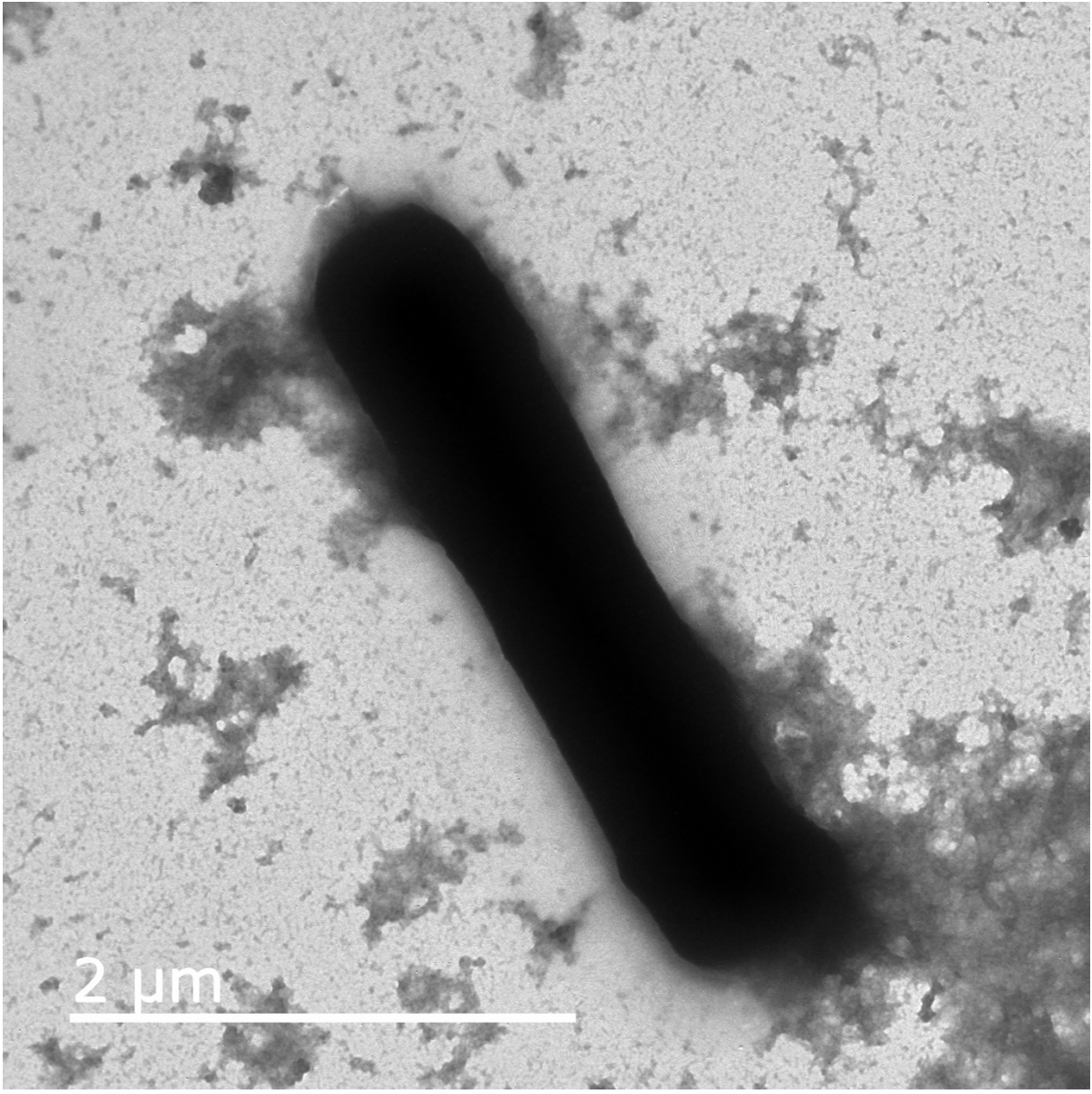

2 μm

Supplement: FIG S2 [file mSphere.00882-20-sf002.pdf]

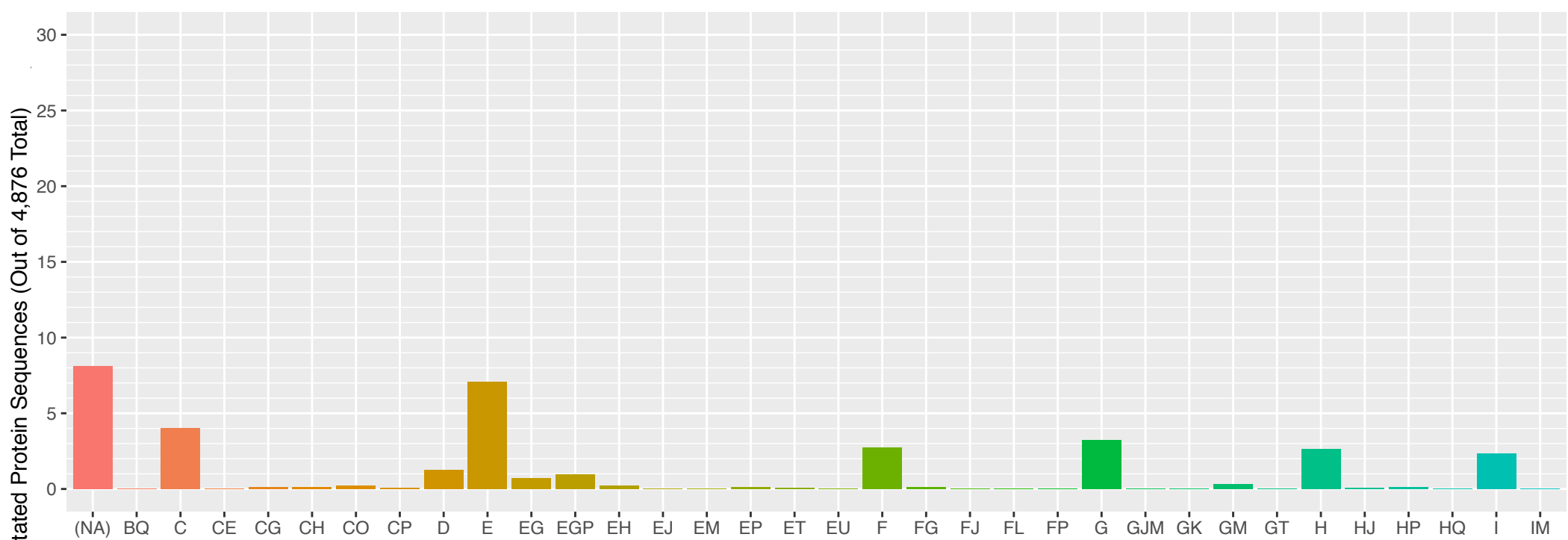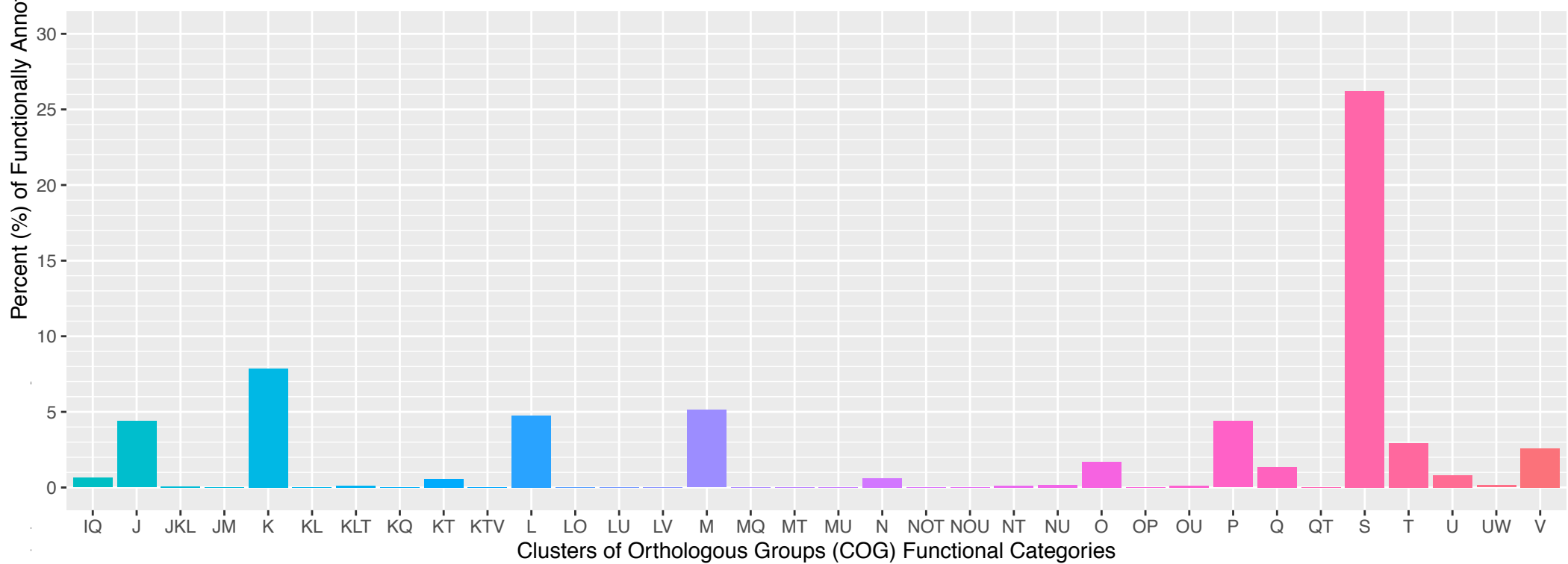

Supplement: FIG S3 [file mSphere.00882-20-sf003.pdf]
